# A novel “7 sutures and 8 knots” surgical technique in reverse shoulder arthroplasty for proximal humeral fractures: tuberosity healing improves short-term clinical results

**DOI:** 10.1186/s10195-023-00697-4

**Published:** 2023-05-08

**Authors:** Elisa Troiano, Giacomo Peri, Irene Calò, Giovanni Battista Colasanti, Nicola Mondanelli, Stefano Giannotti

**Affiliations:** 1 Department of Medicine Surgery and Neurosciences, University of Siena, Siena, Italy; 2 Section of Orthopedics, Azienda Ospedaliero-Universitaria Senese, Policlinico Santa Maria Alle Scotte, Viale Mario Bracci 16, 53100 Siena, Italy

**Keywords:** Reverse shoulder arthroplasty, Tuberosity reconstruction, Tuberosity repair, Proximal humeral fracture, Surgical technique, Fragility fracture

## Abstract

**Background:**

Complex proximal humeral fractures (cPHFs) represent an important public health concern, and reverse shoulder arthroplasty (RSA) has emerged as a feasible treatment option in the elderly with high functional demands. Recent studies have shown that tuberosity healing leads to better clinical outcomes and an improved range of motion. However, the best surgical technique for the management of the tuberosities is still a topic of debate. The purpose of this retrospective observational study is to report the radiographic and clinical outcomes of a consecutive series of patients who underwent RSA for cPHFs using a novel “7 sutures and 8 knots” technique.

**Materials and methods:**

A consecutive series of 32 patients (33 shoulders) were treated with this technique by a single surgeon from January 2017 to September 2021. Results at a minimum follow-up of 12 months and a mean ± SD follow-up of 35.9 ± 16.2 (range 12–64) months are reported.

**Results:**

The tuberosity union rate was 87.9% (29 out of 33 shoulders), the mean Constant score was 66.7 ± 20.5 (range 29–100) points, and the mean DASH score was 33.4 ± 22.6 (range 2–85) points.

**Conclusions:**

The “7 sutures and 8 knots” technique, which relies on three sutures around the implant and five bridging sutures between the tuberosities, is a relatively simple procedure which provides a reliable means for anatomic restoration of the tuberosities and allows functional recovery of the shoulder in elderly patients with cPHFs treated with RSA.

*Level of evidence*: IV; retrospective atudy.

*Trial registration*: At our institution, no institutional review board nor ethical committee approval is necessary for retrospective studies.

## Introduction

Proximal humeral fractures (PHFs) are the seventh most commonly observed fractures in adults and account for 4–10% of all fracture types. A bimodal distribution has been described: PHFs occur in elderly patients with decreased bone strength after low-energy traumas, while most high-energy injuries involve patients under the age of 55 [1]. PHF incidence is rising in the elderly, especially in women, and it now constitutes the third most common osteoporotic fracture [2–4]. The choice of the most effective treatment option for PHFs should take into account the fracture morphology, patient co-morbidities and functional expectations, and it should aim to achieve a pain-free functional shoulder [2, 5]. Also, since PHFs in the elderly are fragility fractures, regardless of the treatment option, a multidisciplinary approach such as a fracture liaison service is fundamental in order to reduce the risk of further fractures [6]. A variety of surgical options can be employed, including closed reduction and percutaneous fixation, closed or open reduction and internal fixation [7], and arthroplasty [3]. Non-operative treatment is generally accepted for undisplaced or minimally displaced PHFs, or for displaced fractures in the elderly with low functional demands or who are not cleared for surgery [3, 4, 8]. The most appropriate treatment for complex PHFs (cPHFs) in the elderly is still a topic of debate, as concomitant osteoporosis and significant comminution prevent the achievement of stable fixation, so they may benefit from arthroplasty rather than osteosynthesis [2, 9, 10]. Historically, hemiarthroplasty (HA) was considered the preferred choice for operative treatment of cPHFs [11, 12]; nevertheless, its outcomes are heterogeneous, so reverse shoulder arthroplasty (RSA) has emerged as an alternative treatment option [12–18]. The main theoretical advantage of RSA is that tuberosity healing and cuff rotator integrity are not prerequisites for a satisfactory outcome since RSA primarily depends on the deltoid muscle to restore shoulder function [3, 14, 15, 17, 19–21]. Nevertheless, it has been shown that tuberosity healing leads to better functional results and active motion, even in RSA [21–26]. This is due to the influence of the volume of the greater tuberosity in restoring the lateral offset, improving the deltoid wrapping over the RSA, and maintaining the function of the subscapularis.  As a result, recent efforts to enhance the tuberosity healing rate have been made [24, 27–35], but a gold standard technique has not been identified.

In the present paper, we present the results of a retrospective observational study conducted on patients older than 65 years of age who underwent RSA for cPHFs with the application of a novel “7 sutures and 8 knots” tuberosity fixation technique to achieve better tuberosity healing.

## Materials and methods

### Study design

A retrospective and observational study was performed. Inclusion criteria were as follows: (1) a cPHF categorized as a Neer three- or four-part fracture, a head-splitting fracture, or with more than 40% of the joint surface head involved; (2) a cPHF occurring in a patient over 65 years of age; (3) a cPHF treated with RSA, a fracture-specific stem, and a standardized novel technique of tuberosity fixation including bone grafting between the metaphyseal part of the stem and the tuberosities performed by a single surgeon; and (4) a minimum clinical and radiological follow-up of 12 months. Patients with previous failed open reduction and internal fixation for PHFs, patients undergoing revision surgery, and patients whose tuberosity comminution did not allow fixation were excluded. At our Institution, no ethical committee nor institutional review board approval is necessary for retrospective studies, and all patients gave their informed consent to data collection and their anonymous use for scientific and teaching purposes.

### Surgical procedure

**Fig. 1:**
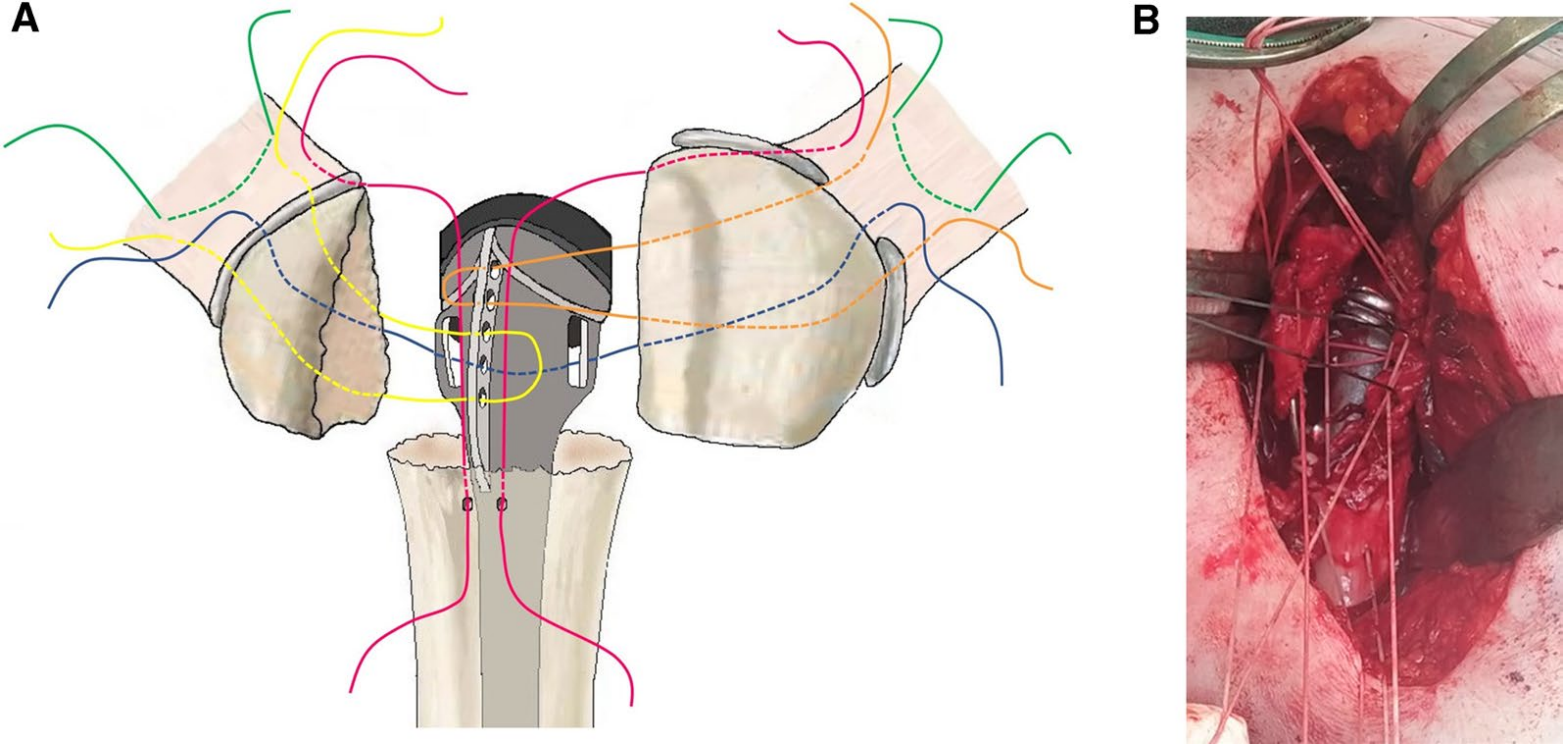
Tuberosity fixation around the prosthetic stem. The relevance of the thread colours is explained in the main text. **A** Graphical illustration of the suture threads. **B** Intra-operative image of the suture threads

**Fig. 2:**
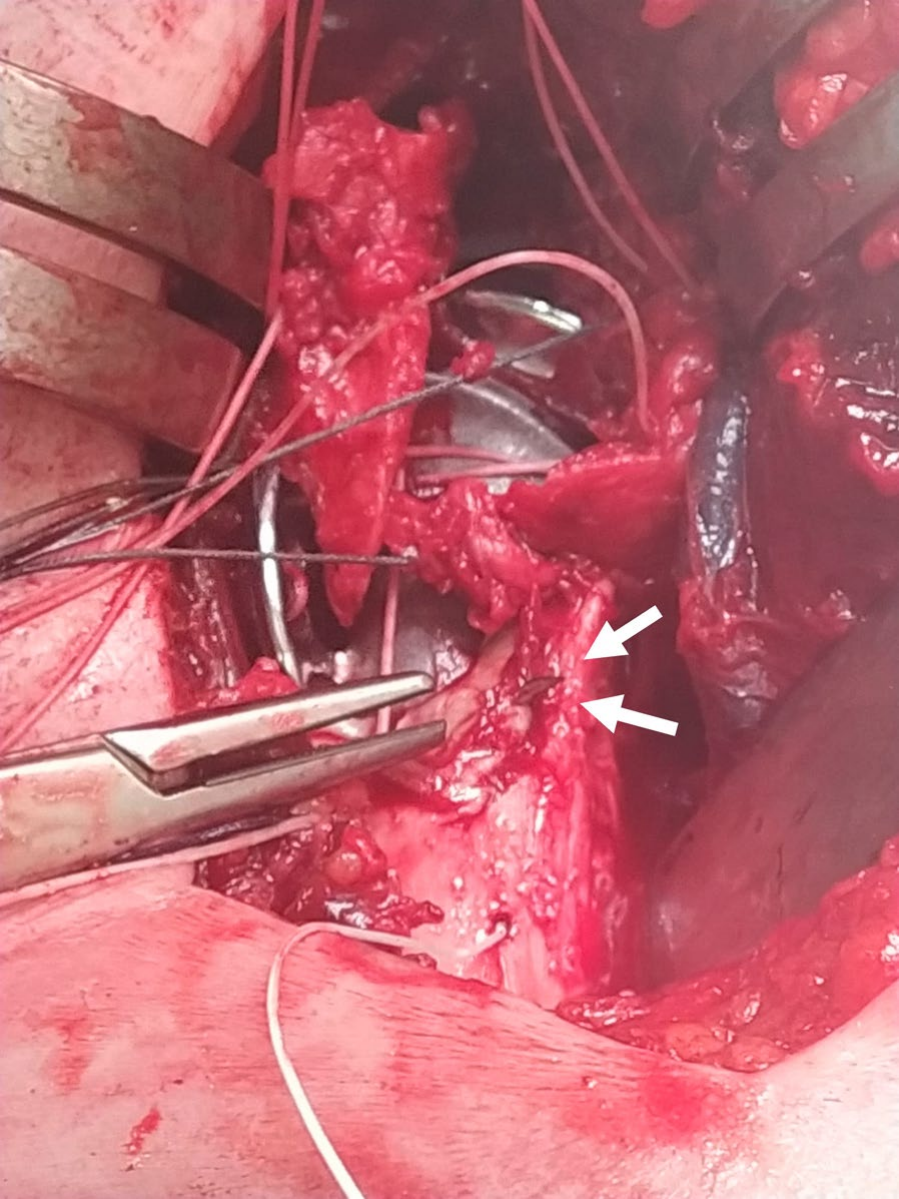
A needle is inserted into the drill hole in the humeral diaphysis before the hardening of the cement to prevent the obstruction of the needle (*white arrows*)

All surgeries were performed by the same senior surgeon with great experience in the RSA procedure performed both for trauma and chronic pathologies. The same prosthesis was implanted in all cases (Equinoxe Reverse-Fracture System Prosthesis; Exactech Inc., Gainesville, FL, USA). All fractures were evaluated by plain radiographs and then further assessed via computed tomography scans with the multiplanar reconstruction technique. A deltopectoral approach was used in all cases. After identifying the fracture planes, the greater and lesser tuberosities were detected and tagged within the context of the tendons (the infraspinatus and teres minor and the subscapularis, respectively) with #2 nonabsorbable sutures (Fig. 1, green threads). The tenotomy of the long head of the biceps brachii tendon was performed. In cases where the supraspinatus tendon was still attached to the greater tuberosity, it was removed, leaving the posterior portion of the rotator cuff intact to facilitate greater tuberosity reduction to the humeral stem during repair. If the bicipital groove was still intact, the tuberosities were separated from each other using a chisel. The glenoid was prepared first after careful retraction of the tuberosities. Reaming of the glenoid surface was performed with a cannulated reamer inserted over a guidewire, and a hole for the central peg was drilled. A standard glenoid baseplate was implanted and secured with the required number of screws, followed by the glenosphere. Whenever possible, pre-operative planning and intraoperative navigation were employed, as previously described [36, 37]. Next, the humeral canal was prepared, and the appropriate fracture stem size was chosen and cemented in 25° of retroversion, being careful to limit the cement to the meta-diaphyseal level. Placing the humeral stem in such retroversion causes the major fin of the stem to be placed at the bicipital groove so that the tuberosity reconstruction can be as anatomical as possible. Before placing the stem, a #2 high-strength suture was passed through the medial fenestration of the prosthesis and around the stem (Fig. 1A, blue thread). Two drill holes were made in the humeral diaphysis before the hardening of the cement, and then two needles were inserted and left in place during cement polymerization to prevent their obstruction (Fig. 2). This expedient is used to avoid possible fragmentation of the cement mantle with subsequent drilling. A #2 high-strength suture was passed into each drill hole and through the superior part of the subscapularis tendon and the external rotator tendon, respectively (Fig. 1A, pink threads). Then, a #2 nonabsorbable suture was passed horizontally through the external rotator tendon, the two cranial holes of the major fin, and again through the tendon (Fig. 1A, orange thread). A similar technique was employed for the subscapularis tendon, engaging the two distal holes of the prosthesis’s major fin (Fig. 1A, yellow thread). Figure 1B shows an intraoperative image of the sutures.

**Fig. 3:**
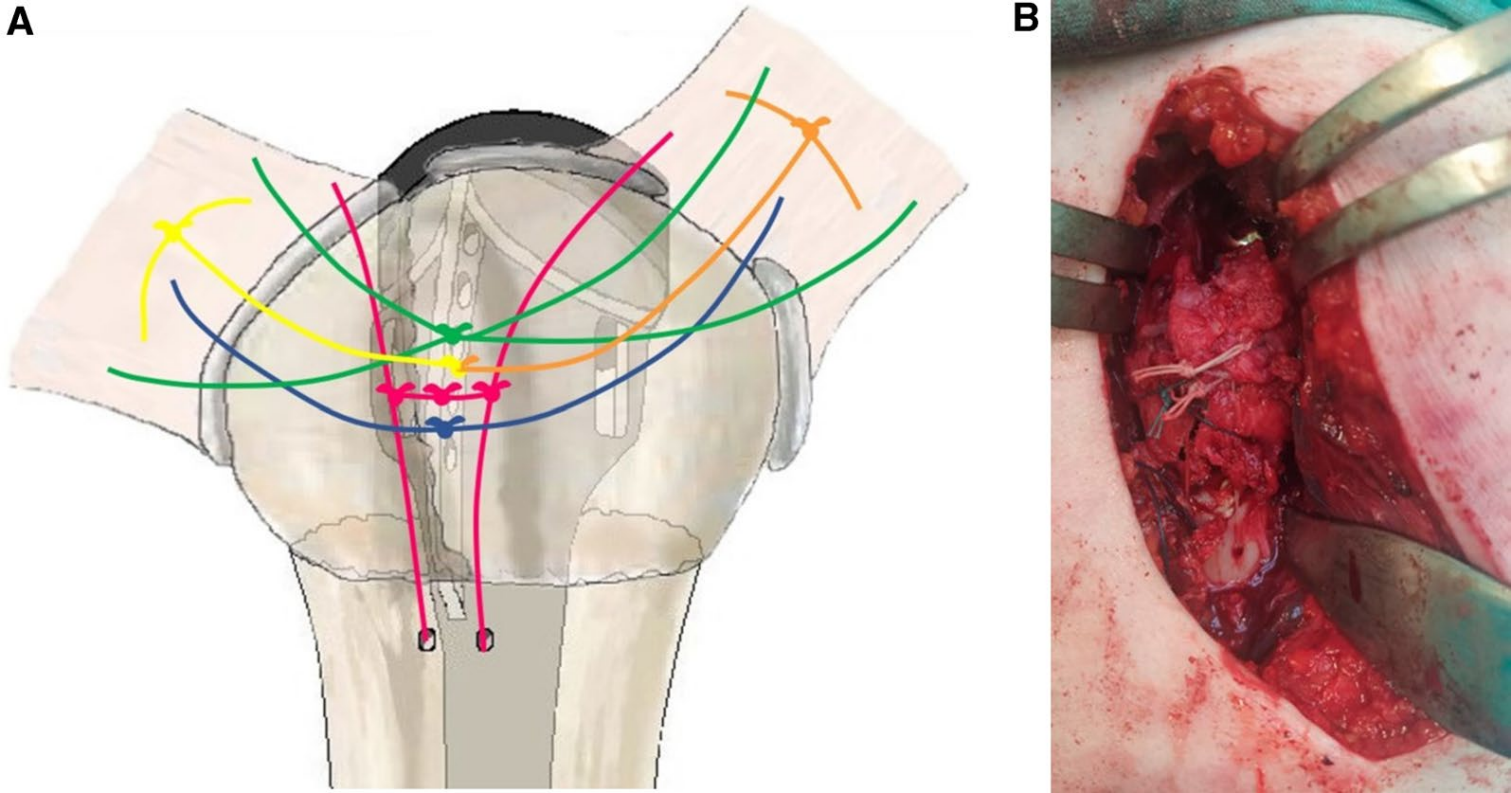
Knotting technique for the tuberosities around the prosthetic stem. The relevance of the thread colours is explained in the main text. **A** Graphical illustration of the knots (the colours correspond to the graphical representation in Fig. 1A). **B** Intra-operative image of the knots

At this point, a cancellous bone graft harvested from the humeral head was placed underneath and next to the major fin of the prosthesis and the tuberosities were secured with eight knots according to the technique illustrated in Fig. 3A, with the seven sutures placed previously. First, the greater and the lesser tuberosities were stabilized on the tuberosity bed (two knots in total, a knot for each tuberosity, yellow and orange threads) and then further tightened together with the medial thread of each suture (one knot). At this point, the two vertical sutures were secured, one for each tuberosity, and then knotted together (three knots in total, pink threads). Thereafter, the sutures used to detect and tag the tuberosities were tied together (a single knot with all four ends of the sutures, green threads). Lastly, the horizontal suture that had been passed through the medial fenestration of the prosthesis and behind the stem was knotted (one knot, blue thread), to further compress the tuberosities onto the stem and onto the humeral shaft (Fig. 3B). To control bleeding, in the absence of contraindications, tranexamic acid was administered both intravenously and locally, as previously described [38]. One suction drain was left in place for 24 h.

### Post-operative care

The same standardized post-operative protocol was used in all patients to minimize possible differences in the functional outcome due to differences in rehabilitation. The arm was rested in a neutral rotation sling in 45° abduction for 4 weeks to minimize tension on the tuberosities and enhance their union. Active and passive range of motion (ROM) of the elbow and the wrist was allowed. The sling was removed at 4 weeks and rehabilitation of the shoulder with a physiotherapist began. Passive ROM exercises in forward elevation and abduction were encouraged at 4 weeks, while active exercises were allowed at 5 weeks. External and internal rotation and strengthening exercises were not allowed until 6 weeks from surgery. No heavy lifting was allowed until 9 weeks post-operatively, and a return to all activities was permitted at 3 months post-operatively.

### Clinical and radiological assessment

All patients were evaluated clinically and radiographically at 1, 3, 6 and 12 months after surgery and then annually. Shoulder function was assessed using the Constant scoring systems [39], and active ROM was recorded in forward elevation, abduction, and external and internal rotation. Overall subjective patient satisfaction was evaluated through a four-grade rating scale (very disappointed, disappointed, satisfied and very satisfied) and the Disability of the Arm, Shoulder and Hand (DASH) scoring system [40]. Plain radiographs in the true antero-posterior view (Grashey projection) of the shoulder with a standardized “shoulder protocol” (65–70 kV, 16 mAs) were obtained at each visit. The greater tuberosity was considered healed when it was visible on the X-rays (Grashey projection in neutral rotation) and fused to the humeral shaft. Glenoid notching was evaluated according to the Nerot–Sirveaux classification [41].

## Results

A series of 32 consecutive patients (33 shoulders) met the inclusion criteria: there were 6 males (18.2%) and 26 females (27 shoulders) (81.8%) with a mean (± standard deviation, SD) age of 77.1 ± 7.3 (range 65–92) years who were evaluated at a mean follow-up of 35.9 ± 16.2 (range 12–64) months after surgery.

Intra-operatively, a standard glenoid baseplate was implanted in each case and secured with a mean of 2.4 ± 0.8 (range 2–5) screws with an average length of 31.4 ± 4.4 (range 22–42) mm. The diameter of the glenosphere was chosen to optimally fit the patient’s anatomy: a 38-mm-diameter glenosphere was used in most of the cases (24 shoulders), a 36-mm-diameter glenosphere was used in smaller subjects (5 shoulders, all females), and a 42-mm-diameter glenosphere was used for larger shoulders (4 males). Post-operative anaemia requiring blood transfusion occurred in 15 patients (46.9%), and a patient suffered from a* Clostridium difficile* infection. Inferior glenoid notching (grade 1) was observed in only one patient. Patients were hospitalized for a mean of 8.0 ± 4.3 (range 3–26) days.

**Fig. 4:**
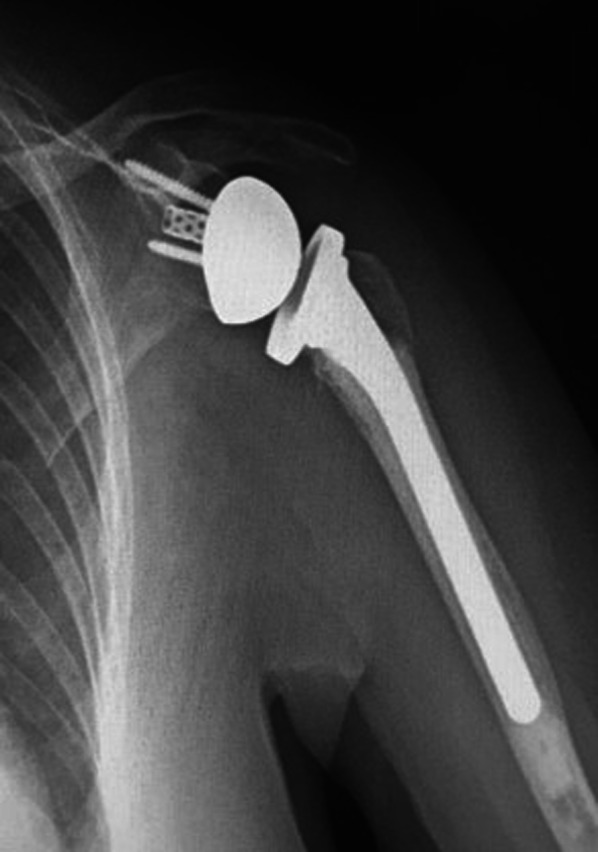
Complete healing of the tuberosities was observed in 85% of the cases at follow-up

The mean ± SD active forward elevation was 129° ± 31° (range 60–180°), the mean abduction was 118° ± 27° (range 70–160°), the mean external rotation was 37° ± 8° (range 23–55°), and the mean internal rotation was 6 ± 3 (range 2–10) points on a converted scale which corresponded to reaching the L1–L3 vertebral level. Only one patient achieved less than 90° of forward elevation. The mean Constant score was 66.7 ± 20.5 (range 29–100) points, and the mean DASH score was 33.4 ± 22.6 (range 2–85) points. At the last follow-up, most patients were satisfied or very satisfied with the results of the surgical procedure. Only three patients (9.1%) were disappointed, and none was very disappointed. Despite the advanced ages of the patients, the use of the “7 sutures and 8 knots” technique plus an autologous bone graft added to a specific reverse shoulder fracture stem resulted in a high tuberosity healing rate and good functional outcomes. Twenty-nine out of 33 shoulders (87.9%) had complete tuberosity healing (Fig. 4), while four patients (12.1%) presented tuberosity resorption. Although not statistically significant, the healed tuberosities group showed better clinical and radiographical results with respect to the non-healed tuberosities group. Overall demographics and functional outcomes for the 33 shoulders are summarized in Table 1, while differences in functional results between the two groups are summarized in Table 2.

**Table 1:** Overall demographics and functional outcomes (33 shoulders)

Demographics	
Male	18.2%
Female	81.8%
Age (years) mean ± SD	77.1 ± 7.3 range (65–92)
Follow-up (months)	
Mean ± SD	35.9 ± 16.2 range (12–64)
Functional outcome (mean ± SD)	
Pain	10.6 ± 3.4 range (4–15)
Activity	15.2 ± 3.8 range (6–20)
ROM	26.8 ± 8.9 range (10–40)
Forward flexion (°)	128.9 ± 30.6 range (60°–180°)
Abduction (°)	118.3 ± 27.3 range (70°–160°)
Extra-rotation (°)	37.5 ± 7.9 range (23°–55°)
Intra-rotation^a^	5.6 ± 2.7 range (2–10)
Power	14.1 ± 7.4 range (2–25)
Constant score	66.7 ± 20.5 range (29–100)
DASH score	33.4 ± 22.6 range (2–85)
Satisfaction with surgery^b^	
Mean ± SD	3.3 ± 0.6 range (2–4)
Perioperative complications	
Blood transfusion	46.9%
Glenoid notching	3%

^a^Points of intra-rotation (hand Dorsum to): buttock = 2; sacro-iliac joint = 4; waist = 6; T12 level = 8; between shoulder blades = 10

^b^Patient satisfaction with the surgery was evaluated through a four-grade rating scale: 1 = very disappointed; 2 = disappointed; 3 = satisfied; 4 = very satisfied

**Table 2:** Functional results according to tuberosity healing

	Healed group	Non-healed group
	87.9%	12.1%
Functional outcome (mean ± SD)		
Pain	10.8 ± 3.4	9.3 ± 2.6
Activity	15.4 ± 3.7	14 ± 4.7
ROM	27.3 ± 8.7	23 ± 11
Forward flexion (°)	131.6 ± 29.1	110 ± 39.2
Abduction (°)	119.1 ± 26.3	112.5 ± 38.6
Extra-rotation (°)	38.1 ± 7.4	33.3 ± 11.6
Intra-rotation^a^	5.9 ± 2.5	4 ± 3.7
Power	14.8 ± 7.4	9 ± 5
Constant score	68.2 ± 20.6	55.3 ± 18.6
DASH score	30.9 ± 20.9	51.6 ± 29.3
Satisfaction with surgery^b^		
Mean ± SD	3.4 ± 0.6	2.5 ± 0.6

^a^Points of intra-rotation (hand Dorsum to): buttock = 2; sacro-iliac joint = 4; waist = 6; T12 level = 8; between shoulder blades = 10

^b^Patient satisfaction with the surgery was evaluated through a four-grade rating scale: 1 = very disappointed; 2 = disappointed; 3 = satisfied; 4 = very satisfied

## Discussion

Anatomical tuberosity healing and rotator cuff integrity have been shown to be essential for good functional recovery after HA for PHFs [41–43]. The unreliable results achieved with HA in the elderly suffering from cPHFs led to attempts to treat these patients with RSA, since the functional outcomes are less dependent on tuberosity healing and cuff integrity [13–15, 19, 20, 44, 45]. However, recent studies have demonstrated that although tuberosity healing is not a prerequisite for a satisfactory outcome after RSA for cPHFs, it still leads to better clinical results [21–27, 46, 47]. It has been proven that tuberosity osteotomy or excision is associated with worse functional results, with particular reference to a loss of external rotation and to a higher risk of RSA instability [27, 48]. The main advantage is better deltoid wrapping, which helps to improve both the function and the stability of the prosthesis [49]. In order to improve the tuberosity healing rate, many surgical techniques have been investigated, but a gold standard reinsertion technique has not been identified [23, 25]. Despite the lack of consensus, it appears from a recent metanalysis [25] that the main fixation method relies on the combination of vertical and horizontal fixation with or without cerclage. Other than the suture techniques and construct, the use of a fracture-specific humeral stem with a large ingrowth surface for tuberosity healing, space for a bone graft and partial cementation techniques could also enhance tuberosity healing [23].

The most pertinent findings of the present study are that tuberosity healing in RSA for cPHFs can be obtained, even in the elderly, by employing a standardized surgical technique, and that tuberosity healing leads to an improved functional outcome and increased patient satisfaction, even if these are not statistically significantly enhanced according to the present data. The pivotal points of this novel surgical technique are the use of a standardized and reproducible tuberosity suture technique, the use of a fracture-specific prosthetic stem associated with an autologous bone graft (harvested from the fractured head), and the application of a partial cementation technique. In our opinion, of paramount importance for obtaining a high tuberosity healing rate is “friendly” tuberosity management, with an optimal balance between the tension and elasticity of the construct. The present “7 sutures and 8 knots” technique employs three high-strength sutures combined with four nonabsorbable sutures and aims to achieve this correct balance, which can favour a high healing rate. In addition, as the geometry of the stem affects the bone integration around it [23, 24, 42], a fracture-specific prosthesis was implanted in all cases. By reducing the proximal metal surface, a larger ingrowth surface is obtained, which can allow better reduction of the tuberosities to the stem and to each other and the use of an autologous bone graft, which may further enhance bone healing [23, 25, 26, 50]. Given the poor bone quality in the elderly, our preference is to cement the stem in all patients. According to previous studies reported in the literature [23, 25, 30, 51], the advantages of cementation include a low rate of iatrogenic fracture, the ability to provide optimal initial stability of the implant and fixation independent from osteogenesis, and the anti-infection ability of the antibiotic-loaded bone cement. However, direct thermal reactions and disturbance of the local blood flow might inhibit tuberosity healing. Accordingly, we limited the cementation to the meta-diaphyseal humeral portion, as suggested by Singh et al. [52].

Despite the advanced age of our study group, fixation of the tuberosities associated with an autologous bone graft and the use of a fracture-specific stem and the partial cementation technique resulted in a high rate of tuberosity healing (> 85%). Our results confirm those of Grubhofer et al. [53], Boileau et al. [24], and Levy and Badman [54], who all observed a similar tuberosity healing rate (> 84%). In the present study, neither shoulder instability nor loosening were reported within the study period, which is consistent with previous studies that also underlined the importance of achieving tuberosity healing to prevent such complications [14, 24].

The present series demonstrates that the restoration of a better active ROM and better subjective results can be expected after tuberosity reconstruction and healing. Among the four patients in whom the tuberosities did not heal, two were disappointed with the results of the surgical procedure and complained about difficulties with activities of daily living (ADLs) which required active forward elevation or rotations. On the other hand, only one patient with healed tuberosities complained about difficulties with ADLs, probably because the dominant shoulder was involved by the fracture and the contralateral one was affected by a severe cuff arthropathy.

This study has limitations. First, it is a retrospective study with a relatively small sample size: it may be difficult to correctly generalize the obtained data. Moreover, the relatively short follow-up could underestimate additional functional improvement or complications beyond 1 year post-operatively. However, no dislocations were observed, which is the primary early complication after RSA for cPHFs (it typically occurs within the first 3 months post-operatively) [22]. Second, this novel technique has been shown to be reproducible, but there may be occurrences where it is not feasible. Extreme tuberosity comminution and therefore a complete lack of bone could prevent the use of such a suturing technique, even though, in our experience, we were not able to employ the “7 sutures and 8 knots” technique in only a single case after we standardized it. On that occasion, we tried to reconstruct the tuberosities with bone cement to guarantee implant stability.

RSA has been demonstrated to be a feasible surgical option to treat cPHFs in the elderly and, although its function relies mainly on the deltoid muscle, reattaching the tuberosities leads to better functional and clinical outcomes. However, there is no consensus regarding the best surgical technique to obtain the highest rate of tuberosity consolidation. The present study shows that the “7 sutures and 8 knots” technique is a relatively straightforward and reproducible method, and, given the results (a tuberosity consolidation rate of > 85%), it is possible to affirm that—despite the above-mentioned limitations—it can provide an excellent success rate, considering both the mean age and the poor bone quality of the study group and the previous results reported in the literature.

## Data Availability

The datasets used and/or analysed during the current study are available from the corresponding author on reasonable request.

## Abbreviations

ADLs: Activities of daily living
cPHFs: Complex proximal humerus fractures
DASH score: Disability of the Arm, Shoulder and Hand score
HA: Hemiarthroplasty
ROM: Range of motion
RSA: Reverse shoulder arthroplasty
SD: Standard deviation
